# Predictive value of metabolic profiling in cardiovascular risk scores: analysis of 75 000 adults in UK Biobank

**DOI:** 10.1136/jech-2023-220801

**Published:** 2023-09-12

**Authors:** Danyao Jin, Eirini Trichia, Nazrul Islam, Sarah Lewington, Ben Lacey

**Affiliations:** 1 Nuffield Department of Population Health (NDPH), University of Oxford, Oxford, UK; 2 MRC Population Health Research Unit, NDPH, University of Oxford, Oxford, UK; 3 Faculty of Medicine, University of Southampton, Southampton, UK

**Keywords:** CARDIOVASCULAR DISEASES, EPIDEMIOLOGY, PRIMARY HEALTH CARE

## Abstract

**Background:**

Metabolic profiling (the extensive measurement of circulating metabolites across multiple biological pathways) is increasingly employed in clinical care. However, there is little evidence on the benefit of metabolic profiling as compared with established atherosclerotic cardiovascular disease (CVD) risk scores.

**Methods:**

UK Biobank is a prospective study of 0.5 million participants, aged 40–69 at recruitment. Analyses were restricted to 74 780 participants with metabolic profiling (measured using nuclear magnetic resonance) and without CVD at baseline. Cox regression was used to compare model performance before and after addition of metabolites to QRISK3 (an established CVD risk score used in primary care in England); analyses derived three models, with metabolites selected by association significance or by employing two different machine learning approaches.

**Results:**

We identified 5097 incident CVD events within the 10-year follow-up. Harrell’s C-index of QRISK3 was 0.750 (95% CI 0.739 to 0.763) for women and 0.706 (95% CI 0.696 to 0.716) for men. Adding selected metabolites did not significantly improve measures of discrimination in women (Harrell’s C-index of three models are 0.759 (0.747 to 0.772), 0.759 (0.746 to 0.770) and 0.759 (0.748 to 0.771), respectively) or men (0.710 (0.701 to 0.720), 0.710 (0.700 to 0.719) and 0.710 (0.701 to 0.719), respectively), and neither did it improve reclassification or calibration.

**Conclusion:**

This large-scale study applied both conventional and machine learning approaches to assess the potential benefit of metabolic profiling to well-established CVD risk scores. However, there was no evidence that metabolic profiling improved CVD risk prediction in this population.

**WHAT IS ALREADY KNOWN ON THIS TOPIC**

- Although previous studies have examined the associations of metabolic biomarkers with incidence and mortality of numerous common diseases, including cardiovascular disease (CVD), there is little evidence on the benefit of metabolic profiling in clinical practice to identify those at high risk of CVD.

**WHAT THIS STUDY ADDS**

- This study found no evidence of substantive improvement in prediction accuracy when adding metabolic profiling to a well-established CVD risk score (with information of cholesterol, blood pressure, body mass index and medical history). This was despite the use of machine learning methods to account for complex interactions of highly correlated metabolites.

**HOW THIS STUDY MIGHT AFFECT RESEARCH, PRACTICE OR POLICY**

- As this prospective study of middle-aged adults from the UK general population found no evidence that metabolic profiling improved CVD risk prediction, it is unlikely that such measures would be valued for CVD prediction in clinical practice (or as part of national screening programmes) in this population, although replication in other populations (or subgroups, such as young adults or the elderly) is warranted.

## Introduction

 Early identification of individuals at risk is important for primary prevention of major atherosclerotic cardiovascular disease (CVD). Several risk assessment algorithms have been developed, including the Framingham Risk Score, Systematic COronary Risk Evaluation (SCORE) and Pooled Cohort Equations.^1^,^3^ Among these established risk scores, QRISK3 is the most widely used across England’s primary health service,^4^ and National Institute for Health and Care Excellence (NICE) are currently recommending that atorvastatin 20 mg is considered for the primary prevention of CVD for people with a QRISK3 score of 10% or more, or with a score less than 10% but with a concern that risk may be underestimated.^5^ However, the discrimination of QRISK3 varies from 0.70 to 0.86 in different UK cohorts, and several studies suggested that QRISK3 may not perform very well in older and multimorbid population.^6^,^8^ Polygenic risk score and lipoprotein(a) have been added to QRISK3 but showed modest improvement in the risk discrimination.^9 10^ Therefore, there is still considerable interest in finding new biomarkers to improve prediction accuracy.

Given the metabolic nature of atherosclerosis, circulating metabolic biomarkers are thought to have great potential to improve risk stratification.^11^ However, current evidence on the predictive value of metabolites has only focused on a limited number of biomarkers with significant linear associations with CVD, which may not reflect the complex pathophysiology of atherosclerosis.^12 13^ Nuclear magnetic resonance (NMR) spectroscopy is a high-throughput technology used for metabolic profiling of numerous metabolites across multiple biological pathways and is being used in large-scale prospective studies.^14^ Therefore, when assessing the predictive value, the large number of metabolites measured through NMR and their complex inter-relations need to be accounted for. Machine learning has been increasingly used for development of prediction models, with the strengths of incorporating highly correlated features and complex interactions that cannot be captured by traditional statistical models.

In this study, we aimed to evaluate whether adding circulating metabolic profiling to a well-established risk score using machine learning methods improved the prediction of 10-year CVD risk.

## Methods

### Study design and population

UK Biobank is a prospective cohort study of approximately 500 000 adults in the United Kingdom recruited from 2006 to 2010.^15 16^ All participants, aged 40–69 at study entry, completed questionnaires and physical measurements and had biological samples collected at recruitment. Ethics approval was given by the North West Multicentre Research Ethics Committee, and the study was conformed to the principles embodied in the Declaration of Helsinki.

### Measurement of metabolic profiling

NMR spectroscopy (Nightingale Health, Finland) was used for metabolic profiling of the baseline plasma samples of 117 980 participants (a random subset of the initial cohort).^17^ To decrease the interference from some unstable biomarkers and to avoid the overfitting due to large number of lipids-related biomarkers, of the metabolites available, the main analyses only included 39 metabolites all measured with comparable validity to clinical chemistry, as the candidate biomarkers (online supplemental table S1).^18^ In the sensitivity analyses, we expanded the candidate metabolites to a larger scope of NMR-derived metabolites that available in the UK Biobank (online supplemental table S2).^18^

### Definition of risk scores

In the main analyses, the metabolites were added to QRISK3, an established risk score widely used across England’s primary health service.^4^ QRISK includes information on age, ethnicity, deprivation, systolic blood pressure (SBP), body mass index (BMI), total cholesterol to high-density lipoprotein (HDL) cholesterol ratio (measured by traditional chemistry method), smoking status, family history of coronary heart disease and medical history of a series of diseases, which were selected based on Bayes information criterion. In the sensitivity analyse, QRISK3 was replaced by SCORE2, which was another algorithm for risk prediction of CVD that widely used in European population, scoring by age, smoking status, SBP and total and HDL cholesterol. Detailed definitions of QRISK3 and SCORE2 variables and mapping in the UK Biobank are provided in online supplemental methods and table S3.

### Ascertainment of incident CVD

Incident CVD was defined as the first-ever coronary heart disease, ischaemic stroke or transient ischaemic attack, identified from Hospital Episode Statistics (including diagnostic codes and relevant procedures) and the Office for National Statistics cause of death data, using codes of the 10th edition of the International Classification of Disease and coronary-related procedures (coronary artery bypass surgery or percutaneous transluminal angioplasty stent placement) by the OPCS Classification of Interventions and Procedures (online supplemental table S4).

### Statistical analysis

The analyses were restricted to participants without prior CVD and those not taking statins at baseline, and further excluded the participants with missing or outlying in QRISK3 variables (online supplemental figure S1). Since the participants in the UK Biobank are overall healthier (with lower incidence of CVD) than the general UK population, QRISK3 score was recalibrated by refitting the baseline survival function to the study population (online supplemental methods).

The candidate metabolites were selected in three ways: (1) adding the metabolites that were significantly associated with CVD (independently from QRISK3 score) to QRISK3; (2) adding all metabolites to QRISK3 and penalised by elastic-net and (3) adding the novel metabolites selected by Boruta SHapley Additive exPlanations (BorutaSHAP) based on Extreme gradient boosting algorithm (XGBoost) to QRISK3. Elastic-net is a regression method that performs regularisation and variable selection simultaneously, with the strength of handling highly correlated variables.^19^ XGBoost is a tree-based machine learning method where new models are created that predict the residuals or errors of prior models and then added together to make the final prediction.^20 21^ It allows for including higher order interactions and accounting for complex non-linear relationships and was chosen as our third model because of its modest computational cost and outstanding performance of risk prediction in recent studies involving a large number of proteins or metabolites.^22 23^ BorutaSHAP is a wrapper feature selection method to explain how much each factor in a model has contributed to the prediction, and the combination with Boruta feature selection algorithm ensures a faster and more stable feature selection.^24^ Detailed explanations of the machine-learning and feature selection methods are provided in online supplemental methods. The hyperparameters were fine tuned using five-fold cross-validation (online supplemental table S5). In all three cases, prediction performance was assessed using Cox proportional hazards’ regression w/o the metabolites. Bootstrapping (500 times) was applied to evaluate the optimism of the models.

Harrell’s C-index was used to assess the discriminatory ability (how the model separate cases from controls) of each model. The improvement in reclassification after adding metabolites was evaluated by the integrated discrimination improvement (IDI) and net reclassification improvement (NRI). IDI summarises the extent that a new model increases risk in events and decreases risk in non-events compared with the old model, while NRI quantifies the appropriateness of the change in predicted probabilities or categorised risk group when changing from old to new model. 10-year probability of event >10% was categorised as high risk and set as the cut-off for categorical NRI. The calibration, measuring how close the predicted probability is to the observed risk, was assessed with calibration plots at 10 years. All analyses followed the suggestions from TRIPOD,^25^ and all models were developed and evaluated separately for men and women in Python 3.9.12.

## Results

After exclusions, 74 780 participants remained, with mean age of 55 years at study entry. The overall baseline characteristics of the study population were similar to the whole UK Biobank population (online supplemental table S6). Among the study population, 44% were men, 10% were current smokers and 41% reported to have family history of heart disease. After a 10-year follow-up, 5097 (6.8%) incident CVD events occurred, with about two times the rate in men than women (9.4% vs 4.8%). Compared with participants who did not have an incident CVD event, those with incident CVD were on average older, with higher BMI, SBP and higher ratio of total cholesterol to HDL cholesterol, and more likely to be men and current moderate/heavy smokers. Participants who experienced CVD during follow-up were also more likely to have family heart disease history and baseline chronic disease history (table 1).

**Table 1:** Characteristics of baseline QRISK factors by 10-year incident CVD

	Incident CVD		All
	No	Yes	
Number of participants	69 683	5097	74 780
Age, sex and socioeconomic factors			
Men, %	42.0	59.8	43.3
Baseline age, years	55.0 (8.0)	59.6 (7.0)	55.3 (8.0)
White, %	94.8	95.7	94.9
Townsend Deprivation Index*	1.5 (2.9)	1.3 (3.0)	1.4 (3.0)
Anthropometry, blood pressure and lipids by clinical chemistry			
Body mass index, kg/m^2^	26.9 (4.5)	27.9 (4.6)	27.0 (4.6)
Systolic blood pressure, mm Hg	136.2 (17.9)	143.7 (18.1)	136.7 (18.4)
SD between two readings,† mm Hg	5.1 (4.0)	5.5 (4.2)	5.2 (4.0)
Total cholesterol to HDL-C ratio	4.2 (1.1)	4.5 (1.2)	4.2 (1.1)
Smoking intensity, %			
Ex-smoker	32.4	35.7	32.7
Light smoker (<10 per day)	4.8	4.9	4.8
Moderate smoker (10–19 per day)	2.9	4.7	3.0
Heavy smoker (≥20 per day)	2.3	4.6	2.4
Family history of heart disease,‡ %	39.5	50.5	40.2
Disease and medication history, %			
Type 1 diabetes	0.3	0.6	0.3
Type 2 diabetes	1.6	3.6	1.7
Chronic kidney disease (stage 3,4,5)	1.5	2.8	1.6
Atrial fibrillation	0.8	2.6	0.9
Migraines	4.5	5.2	4.6
Rheumatoid arthritis	1.1	2.4	1.2
Systemic lupus erythematosus	0.1	0.3	0.1
Severe mental illness§	5.0	5.5	5.0
Erectile dysfunction	0.2	0.5	0.2
Hypertension treatment	11.6	23.0	12.3
Atypical antipsychotic medication	0.2	0.2	0.2
Regular steroid tablets	0.7	1.8	0.8

Sex -adjusted characteristics of QRISK factors at baseline by 10-year incident ASCVD. Incident CVD defined as the first-ever coronary heart disease, ischaemic stroke or transient ischaemic attack. Continuous variables are presented as mean (standard deviationSD) and categorical variables are presented as column percentages.

* Higher values indicate higher levels of material deprivation.

† QRISK asks for standard deviation of systolic blood pressure values recorded in the 5 years before study entry, but UK Biobank only provided two automated or manual readings at study entry.

‡ QRISK asks for the family history in first-degree relatives aged less than 60 years, but UK Biobank only identified family history in first degree relatives in all ages.

§ Includes schizophrenia, bipolar disorder and moderate/severe depression.

CVDcardiovascular diseaseHDL-Chigh-density lipoproteins cholesterol

The HR of the recalibrated QRISK3 score was 1.17 (95% CI 1.15 to 1.18) per one point higher in women and 1.08 (1.07 to 1.09) in men. Independently from QRISK3 score, 12 metabolites (HDL cholesterol, two apolipoprotein biomarkers, six fatty acid ratio biomarkers, histidine, albumin and glycoprotein acetyls) in women and 5 (very low-density lipoprotein cholesterol, apolipoproteinB (ApoB) to ApolipoproteinA-1 (ApoA-1) ratio, omega-3 fatty acid concentration and its ratio to total fatty acids, albumin and glycoprotein acetyls) in men remained significantly associated with CVD (table 2). In the two machine learning models of both sexes, fewer fatty acids were selected, but some amino acids and glycolysis-related metabolites were included as predictors. Compared with the selection criteria by association significance (first model), albumin and glycoprotein acetyls were also selected by the two machine learning models for both sexes, while total triglycerides in women and glycine and leucine in men were newly selected as novel metabolites by the two machine-learning models (online supplemental table S7).

**Table 2:** Associations of clinical metabolites independent from QRISK3 score

	Hazard ratio (95% CI)	
	Women	Men
Recalibrated QRISK3 score	1.17 (1.15 to 1.18)*	1.08 (1.07 to 1.09)*
Cholesterols and triglycerides		
Total cholesterol	0.96 (0.91 to 1.00)	1.01 (0.97 to 1.05)
VLDL cholesterol	1.04 (0.99 to 1.08)	1.01 (0.97 to 1.05)
LDL cholesterol	0.98 (0.94 to 1.03)	1.01 (0.98 to 1.05)
HDL cholesterol	0.89 (0.85 to 0.93)*	0.98 (0.94 to 1.02)
Total triglycerides	1.02 (0.98 to 1.07)	0.97 (0.94 to 1.02)
Fatty acids		
Total fatty acids	1.01 (0.96 to 1.05)	0.98 (0.95 to 1.02)
Omega-3 fatty acids	0.96 (0.92 to 1.00)	0.94 (0.91 to 0.97)*
Omega-6 fatty acids	0.97 (0.93 to 1.01)	1.00 (0.96 to 1.04)
Polyunsaturated fatty acids	0.96 (0.92 to 1.01)	0.98 (0.95 to 1.02)
Monounsaturated fatty acids	1.05 (1.00 to 1.09)	0.98 (0.95 to 1.02)
Saturated fatty acids	1.01 (0.97 to 1.06)	0.98 (0.95 to 1.02)
Docosahexenoic acid	0.95 (0.91 to 0.99)	0.95 (0.92 to 0.98)
Linoleic acid	0.96 (0.92 to 1.00)	1.00 (0.96 to 1.03)
Omega-3 to total fatty acids	0.95 (0.91 to 0.99)	0.94 (0.90 to 0.97)*
Omega-6 to total fatty acids	0.93 (0.89 to 0.97)*	1.03 (0.99 to 1.07)
Polyunsaturated to total fatty acids	0.92 (0.88 to 0.95)*	1.01 (0.97 to 1.05)
Monounsaturated to total fatty acids	1.13 (1.08 to 1.18)*	1.00 (0.96 to 1.04)
Saturated to total fatty acids	1.02 (0.98 to 1.06)	0.99 (0.96 to 1.03)
Docosahexaenoic acid to total fatty acids	0.94 (0.89 to 0.98)*	0.96 (0.92 to 0.99)
Linoleic acid to total fatty acids	0.92 (0.88 to 0.96)*	1.02 (0.99 to 1.06)
Polyunsaturated to monounsaturated fatty acids	0.88 (0.84 to 0.92)*	1.00 (0.96 to 1.04)
Omega-6 to omega-3 fatty acids	1.02 (0.98 to 1.07)	1.04 (1.01 to 1.08)
Apolipoproteins		
Apolipoprotein B	1.02 (0.97 to 1.06)	1.02 (0.99 to 1.06)
Apolipoprotein A-1	0.91 (0.87 to 0.95)*	0.96 (0.93 to 1.00)
Apolipoprotein B to apolipoproteinA-1	1.07 (1.02 to 1.12)*	1.06 (1.02 to 1.10)*
Amino acids		
Alanine	1.02 (0.98 to 1.07)	0.98 (0.94 to 1.01)
Glycine	0.95 (0.91 to 0.99)	0.96 (0.92 to 0.99)
Histidine	0.91 (0.87 to 0.95)*	0.97 (0.93 to 1.00)
Isoleucine	1.02 (0.98 to 1.06)	1.01 (0.98 to 1.05)
Leucine	1.00 (0.96 to 1.05)	1.00 (0.97 to 1.04)
Valine	0.99 (0.95 to 1.03)	0.98 (0.95 to 1.02)
Total branched-chain amino acids	1.00 (0.96 to 1.04)	0.99 (0.96 to 1.03)
Phenylalanine	1.05 (1.01 to 1.09)	1.04 (1.01 to 1.08)
Tyrosine	1.00 (0.96 to 1.05)	1.01 (0.97 to 1.04)
Glycolysis-related metabolites		
Glucose	1.02 (0.98 to 1.06)	1.01 (0.98 to 1.04)
Lactate	1.03 (0.99 to 1.08)	0.99 (0.95 to 1.02)
Fluid balance		
Creatinine	1.02 (0.98 to 1.06)	1.01 (0.98 to 1.04)
Albumin	0.88 (0.84 to 0.92)*	0.91 (0.88 to 0.94)*
Inflammation		
Glycoprotein acetyls	1.14 (1.09 to 1.19)*	1.06 (1.02 to 1.10)*

(HR) per one score higher of concentration. HR of each metabolite was calculated by Cox proportional-hazards regression with adjustment of QRISK3 score.

* Associations remained significant (p-value<0.01) by correction of false discovery rate using Benjamini-Hochberg method., which is also marked as boldface in the table.

HDLhigh-density lipoproteinLDLlow-density lipoproteinVLDLvery LDL

**Figure 1:**
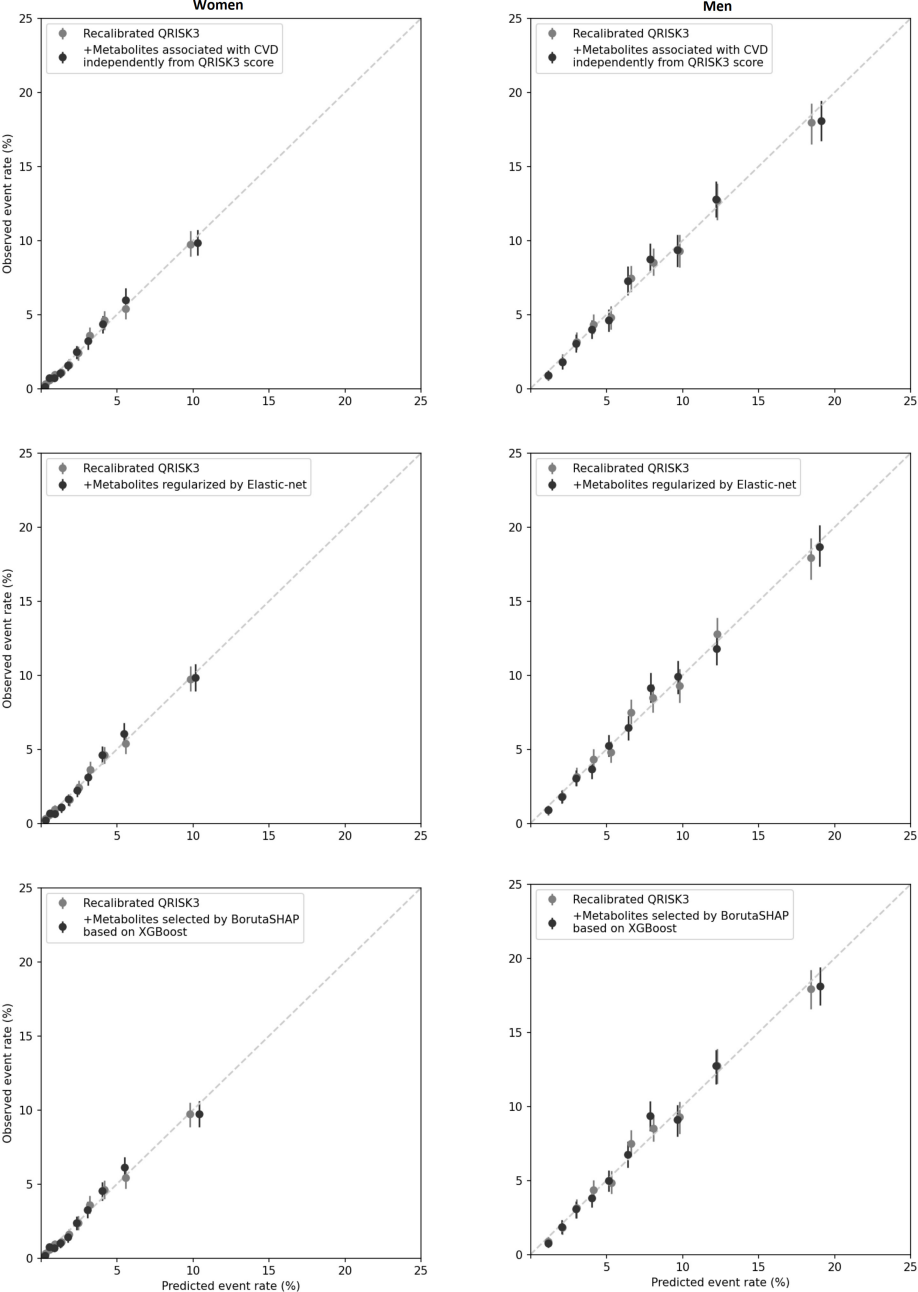
Calibration of risk prediction models for 10-year CVD risk. For each model, the observed and predicted CVD event rates are shown for each of 10 equally sized groups of absolute predicted risk. Vertical lines represent 95% CIs (bootstrap percentile CI, bootstrap for 500 times). CVD, cardiovascular disease.

Harrell’s C-index of the recalibrated QRISK3 was 0.750 (95% CI 0.739 to 0.763) for women and 0.706 (95% CI 0.696 to 0.716) for men (table 3). Adding metabolites to QRISK, in all three models, did not improve the discrimination in women (C-index of three models are 0.759 (0.747 to 0.772), 0.759 (0.746 to 0.770) and 0.759 (0.748 to 0.771), respectively) or men (0.710 (0.701 to 0.720), 0.710 (0.700 to 0.719) and 0.710 (0.701 to 0.719), respectively). The reclassification showed no improvement after adding the metabolites, with statistically significant relative IDI, but less than 0.5% in all three models of both sexes. Although the continuous NRI showed statistically significant increase in most models, the categorical NRI (setting 10-year event probability ≥10% as high risk), which is a better measure of reclassification, showed no improvement in either men or women. Calibration plots did not show any significant change either (figure 1).

**Table 3:** Comparing prediction performance of 10-year CVD risk w/o metabolites

Prediction performance	Women (95% CI*)	Men (95% CI)
**Recalibrated QRISK3**		
Harrell’s C-index†	0.750 (0.739 to 0.763)	0.706 (0.696 to 0.716)
**Adding metabolites associated with CVD independently from QRISK3 score**		
C-statistics	0.759 (0.747 to 0.772)	0.710 (0.701 to 0.720)
IDI‡ (%)	0.30 (0.17 to 0.41)	0.20 (0.12 to 0.28)
Continuous NRI§ (%)	12.4 (6.7 to 16.6)	6.8 (2.7 to11.6)
Events	6.5 (1.0 to 10.8)	4.0 (0.0 to 8.3)
Non-events	5.9 (5.0 to 6.8)	2.8 (1.8 to 3.9)
Categorical NRI (%)	0.3 (−1.8 to 0.9)	0.9 (−0.2 to 2.0)
Events	0.4 (−1.2 to 1.5)	0.4 (−0.7 to 1.4)
Non-events	0.7 (−0.8 to 0.5)	0.5 (0.3 to 0.8)
**Adding metabolites withregularisation(using Elastic-net)**		
Harrell’s C-index	0.759 (0.746 to 0.770)	0.710 (0.700 to 0.719)
IDI (%)	0.16 (0.03 to 0.26)	0.16 (0.04 to 0.25)
Continuous NRI (%)	4.4 (−0.7 to 9.6)	7.4 (3.3 to 11.0)
Events	4.7 (−0.3 to 9.9)	5.2 (1.4 to 8.8)
Non-events	0.3 (−1.3 to 0.7)	2.2 (1.2 to 3.3)
Categorical NRI (%)	0.3 (−1.6 to 1.1)	0.7 (−0.8 to 1.8)
Events	0.2 (−1.2 to 1.5)	0.3 (−1.1 to 1.5)
Non-events	0.4 (−0.5 to 0.3)	0.4 (0.1 to 0.7)
Adding metabolites selected by BorutaSHAP from XGBoost		
Harrell’s C-index	0.759 (0.748 to 0.771)	0.710 (0.701 to 0.719)
IDI (%)	0.26 (0.11 to 0.38)	0.13 (0.03 to 0.20)
Continuous NRI (%)	14.7 (9.2 to 19.7)	5.5 (1.7 to 9.5)
Events	2.7 (−2.9 to 7.7)	0.1 (−4.0 to 3.4)
Non-events	12.0 (11.0 to 12.9)	5.9 (4.9 to 6.9)
Categorical NRI (%)	0.0 (−1.6 to 1.3)	0.7 (−0.5 to 1.8)
Events	0.6 (−0.9 to 1.9)	0.3 (−0.9 to 1.2)
Non-events	0.6 (−0.7 to 0.5)	0.5 (0.2 to 0.7)

Comparing prediction performance of 10-year CVD risk w/o metabolites. In all models, metabolites are added to recalibrated QRISK3 using Cox proportional-hazards regression. Hyper-parameters of each model are in appendix.

* Bootstrap percentile CI, bootstrap for 500 times.

† Harrell’s C-index, measuring the probability that a randomly selected subject with shorter time-to-event will have a higher predicted probability of event than a randomly selected subject with longer time-to-event.

‡ Integrated discrimination improvement, summarising the extent a new model increases risk in events and decreases risk in non-event compared with the old model.

§ Net reclassification improvement, quantifying the appropriateness of the change in predicted probabilities or categorised risk group when changing from old to new model; categorical NRI is based on a 10% risk threshold.

CVDcardiovascular diseaseNRInet reclassification improvement

The HRs (per one point higher) of the recalibrated SCORE2 were 1.12 (1.10 to 1.13) in women and 1.07 (1.06 to 1.07) in men (online supplemental table S8). Replacing QRISK3 by SCORE2 had limited impact on the selection of novel metabolites in all three models, of which XGBoost selected the exactly same metabolites as using QRISK3 as the basic score (online supplemental table S9). Meanwhile, adding metabolites to SCORE2 did not significantly improve the overall prediction accuracy, although some slight improvements were observed in continuous NRI, which may largely due to the poorer performance of SCORE2 in the study population (Harrell’s C-index of SCORE2 were 0.731 (0.718 to 0.744) in women and 0.689 (0.679 to 0.699) in men) (online supplemental table S10 and figure S2). Similarly, there was no evidence of prediction improvement when expanding the scope of the candidate metabolites (online supplemental table S11 and figure S3). Among individuals who currently identified as low-risk (10-year predicted risk less than 10%), risk categorisation (measured by categorical NRI) after adding metabolites to QRISK3 showed no improvement in women and limited improvement (less than 6%) in men.

## Discussion

This large-scale prospective study examined the predictive value of adding high-throughput metabolic profiling to an established risk score among 75 000 participants in UK Biobank. To our knowledge, this is the first study to assess the additional predictive value of high-throughput circulating metabolites to a well-established CVD risk score. The application of machine learning approaches allows for highly correlated variables and accounts for the complex interactions between metabolites in atherosclerosis. However, compared with the standard QRISK3 score, there was no evidence of substantive improvement in prediction of 10-year risk of CVD after adding the metabolic biomarkers.

Several previous studies have examined the value of metabolic profiling measured by NMR for the prediction of cardiovascular event or subclinical atherosclerosis.^12 13 26^ Two of these studies, both of which used traditional statistical algorithms, found moderate improvement in discrimination or reclassification, but neither included BMI as an established risk factor in the basic models. One other recent study used risk factors, including BMI in the basic model, and observed very slight C-index improvement of coronary heart disease prediction (0.003 (0.001 to 0.004)) and no improvement of cerebral stroke prediction (0.001 (−0.003 to 0.005)) when adding metabolomics.^26^ However, the basic model of this study still lacked detailed information on several major risk factors, such as family history of heart disease. By contrast, QRISK3 is a score developed from more comprehensive risk factors, including BMI, cholesterol level, family history and aspects of medical history and mediations. Similarly, when using the SCORE2 (a risk score not including BMI and medical history as risk factors) as the basic score in our sensitivity, adding metabolites showed a slight improvement in continuous NRI due to the poorer performance of the original SCORE2; however, the overall prediction accuracy that measured by C-index was not significantly improved.

Two other cohorts have examined the predictive value of metabolites measured by mass spectrometry,^27 28^ which is another type of high-throughput technique for metabolic profiling with the capability of detecting thousands of metabolites.^29^ One study used traditional statistical algorithms and the other applied elastic-net and principal components analysis, and they both observed modest improvement in the prediction of coronary heart disease or subclinical CVD. However, similar as the previous evidence on NMR-derived metabolites, neither of the studies compared the prediction performance with any established risk score. Moreover, because mass spectrometry is more expensive and time-consuming than NMR, the sample size of both studies was relatively small (less than 3000 individuals).

As a result of selecting metabolites that were associated with CVD independently from the QRISK score, our study identified novel potential predictors for cardiovascular risk by using two different machine learning algorithms. Elastic-net allows for handling highly correlated variables and enhances the prediction accuracy by regularisation, while XGBoost is a novel tree-based model with the strength of incorporating complex variables’ interactions that cannot be captured by traditional statistics model. Additionally, BorutaSHAP is a relatively stable feature selection algorithm using shapely value, which provides another way of measuring feature importance other than association. Although prediction performance was not improved in our results, applying machine learning algorithms gave insight into the predictive value of some amino acids and glycolysis-related metabolites that have previously been overlooked in association analyses under linear assumption, and such selection was proved to be robust because most of the metabolites remained to be select as novel biomarkers when changing to use SCORE2 as the basic score in the sensitivity analyses.

This study has a number of key strengths. It uses large-scale metabolite profiling and applies machine learning algorithms. The linkage to NHS electronic health records and national death registries limited loss to follow-up and allowed reliable ascertainment of CVD events. In addition, the use of different analytical methods with different assumptions showed that our results were robust against different assumptions. However, as about 95% of participants are white in the UK Biobank, it is difficult to generalise our results to other ethnicities; more studies are needed in diverse populations and with longer follow-up to compare with other 10-year or life-time risk scores. Furthermore, the UK Biobank is generally healthier than the wider UK population and only included participants aged 40–69. Future analyses should assess the benefit of metabolic profiling to cardiovascular risk in wider age range, in non-white and high-risk individuals, and explore the predictive value of other types of metabolites (eg, gut microbiome).

## Conclusion

This large-scale prospective study provides evidence that compared with an established risk score with information on BMI and medical history, adding circulating metabolic profiling measured by NMR spectroscopy is unlikely to lead to a substantive improvement in CVD risk prediction in primary care.

## supplementary material

10.1136/jech-2023-220801 online supplemental file 1

## Data Availability

Data are available upon reasonable request.
