# Correction: *Predictive value of metabolic profiling in cardiovascular risk scores: analysis of 75 000 adults in UK Biobank*

**DOI:** 10.1136/jech-2023-220801corr1

**Published:** 2024-02-09

Jin D, Trichia E, Islam N, *et al.* Predictive value of metabolic profiling in cardiovascular risk scores: analysis of 75 000 adults in UK Biobank. *J Epidemiol Community Health* 2023;**77**:802–808.

This article has been corrected since it first published – it is now open access under a CC BY licence.

