# Supplementary material for: Predictive value of metabolic profiling in cardiovascular risk scores: analysis of 75 000 adults in UK Biobank
Source: J Epidemiol Community Health. 2023 Sep 12;77(12):e220801. doi: 10.1136/jech-2023-220801 (PMC11418003; doi:10.1136/jech-2023-220801)
Supplement: online supplemental file 1 [file jech-77-12-s001.pdf]

Predictive value of metabolic profiling in cardiovascular risk scores: analysis of 75,000 adults in UK Biobank

Supplementary Appendix

Danyao Jin, MSc[1*]; Eirini Trichia, PhD[1,2*]; Nazrul Islam, PhD[1,3];
Sarah Lewington, DPhil[1,2+]; Ben Lacey, DPhil[1,4+]

Table of Contents

Supplementary Methods 2
QRISK3 variables and mapping in UK Biobank 2
SCORE2 variables[3] 3
Recalibration of QRISK3 and SCORE2 4
Elastic-net 5
XGBoost 6
BorutaSHAP 7
Assessment of prediction performance 8
Reference 9
Table S1: List of clinically validated metabolites for main analyses 10
Table S2: List of metabolites for sensitivity analyses 11
Table S3: Disease and medication codes of QRISK3 variables in UK biobank 12
Table S4: ICD-10 and operation code of cardiovascular disease 13
Figure S1: Flowchart of exclusion criteria for study population in UK Biobank 15
Table S5: Fine-tuning of hyper-parameters (QRISK3) 16
Table S6: Baseline characteristics in UK Biobank versus in study population 17
Table S7: List of selected novel metabolites by different methods (QRISK3) 18
Table S8. Associations of clinical metabolites independent from SCORE2 19
Table S9: List of selected metabolites using different methods (SCORE2) 20
Table S10: Comparing prediction performance of 10-year CVD risk w/o metabolites (SCORE2) 21
Figure S2: Calibration of risk prediction models for 10-year CVD risk (SCORE2) 22
Table S11: Prediction performance of 10-year ASCVD risk w/o metabolites (QRISK3 and wider scope of candidate metabolites) 23
Figure S3: Calibration of risk prediction models for 10-year ASCVD risk (QRISK3 and wider scope of candidate metabolites) 24

Supplementary Methods

QRISK3 variables and mapping in UK Biobank[1,2]

- Age at study entry (years)
- Ethnic origin (White or not state; Indian; Pakistani; Bangladeshi; Other Asian; Black Caribbean; Black African; Chinese; Other ethnic group): *our study only included White participants for analyses*
- Deprivation (as measured by the Townsend score, where higher values indicate higher levels of material deprivation)
- Systolic blood pressure (SBP) (mmHg)
- Measure of systolic blood pressure variability (standard deviation of repeated measures): *UK Biobank does not include information on variability in SBP. Our study derived this variable by the standard deviation between two automated or manual SBP readings at baseline (Variable ID 4080 and 93).*
- Body mass index (kg/m^2)
- Total cholesterol-to-high density lipoprotein cholesterol ratio
- Smoking status (non-smoker, former smoker, light smoker (1-9/day), moderate smoker (10-19/day), or heavy smoker (≥20/day)):
- Family history of coronary heart disease in a first-degree relative aged less than 60 years: *UK Biobank includes illnesses in father (Variable ID 20107), illnesses in mother (Variable ID 20110), and illnesses of siblings (Variable ID 20111), but does not have information on age at diagnosis. Our study assumed age less than 60 years at diagnosis.*
- Diabetes (type 1, type 2, or no diabetes)
- Treated hypertension (diagnosis of hypertension and treatment with at least one antihypertensive drug)
- Rheumatoid arthritis (diagnosis of rheumatoid arthritis, Felty's syndrome, Caplan's syndrome, adult onset Still's disease, or inflammatory polyarthropathy not otherwise specified)
- Atrial fibrillation (including atrial fibrillation, atrial flutter, and paroxysmal atrial fibrillation)
- Chronic kidney disease (stage 3, 4 or 5) and major chronic renal disease (including nephrotic syndrome, chronic glomerulonephritis, chronic pyelonephritis, renal dialysis, and renal transplant)
- Diagnosis of migraine (including classic migraine, atypical migraine, abdominal migraine, cluster headaches, basilar migraine, hemiplegic migraine, and migraine with or without aura)
- Corticosteroid use (including oral or parenteral prednisolone, betamethasone, cortisone, depo-medrone, dexamethasone, deflazacort, efcortesol, hydrocortisone, methylprednisolone, or triamcinolone)
- Systemic lupus erythematosus (including diagnosis of SLE, disseminated lupus erythematosus, or Libman-Sacks disease)
- Second generation "atypical" antipsychotic use (including amisulpride, aripiprazole, clozapine, lurasidone, olanzapine, paliperidone, quetiapine, risperidone, sertindole, or zotepine)
- Diagnosis of severe mental illness (including psychosis, schizophrenia, or bipolar affective disease)
- Diagnosis of erectile dysfunction or treatment for erectile dysfunction (including alprostadil, phosphodiesterase type 5 inhibitors, papaverine, or phentolamine)

SCORE2 variables[3]

- Age at study entry (years)
- Smoking (current vs. other)
- Systolic blood pressure (SBP) (mmHg)
- Diabetes (yes or no)
- Total cholesterol (mmol/L)
- HDL cholesterol (mmol/L)
- Smoking x age interaction
- SBP x age interaction
- Total cholesterol x age interaction
- HDL cholesterol x age interaction
- Diabetes x age interaction

Recalibration of QRISK3 and SCORE2

The participants in UK Biobank are in overall healthier than the general UK population, with lower incidence of CVD in both men and women, and the calibration plot also showed that the original QRISK3 score was overestimated and original SCORE2 was underestimated when applying to the study population (Figure below). Therefore, following TRIPOD guidelines,[4] our study only used the predicted hazard ratios calculated by the original algorithm[5], and refitted the baseline survival function from the study population to obtain recalibrated predicted probabilities. After refitting the baseline risk, the recalibrated predicted risk from QRISK3 and SCORE2 was well calibrated to the observed risk of each individual (in main Figure and Figure S2, respectively).

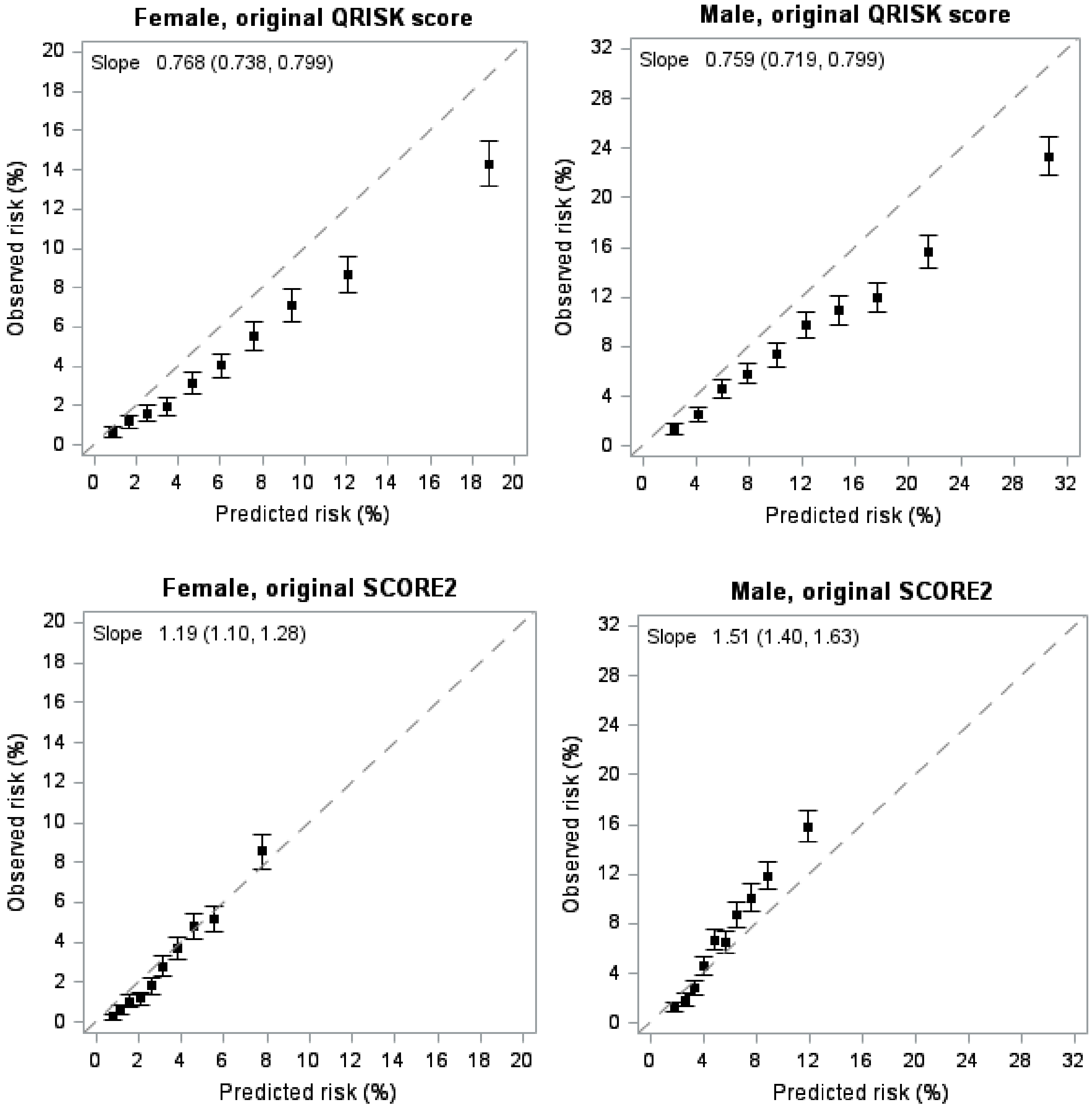

Elastic-net[6,7]

Elastic-net is a regularization and variable selection method that linearly combines the L1 and L2 penalties in the regression model. The method overcomes the limitations of the LASSO when dealing with highly correlated variables.

In our study, elastic-net was applied in Cox proportional hazards model, using the Python package of *sksurv.linear_model.CoxnetSurvivalAnalysis*.[8] The key parameters include:

- n_alphas (int, default: 100) – Number of alphas along the regularization path.
- alphas (array-like or None) – List of alphas where to compute the models.
- alpha_min_ratio (float or "auto", default: "auto") –Determines minimum alpha of the regularization path if alphas is None. The smallest value for alpha is computed as the fraction of the data derived maximum alpha (i.e. the smallest value for which all coefficients are zero).If set to “auto”, the value will depend on the sample size relative to the number of features. If n_samples > n_features, the default value is 0.0001 If n_samples <= n_features, 0.01 is the default value.
- l1_ratio (float, default: 0.5) – The ElasticNet mixing parameter, with 0 < l1_ratio <= 1. For l1_ratio = 0 the penalty is an L2 penalty. For l1_ratio = 1 it is an L1 penalty. For 0 < l1_ratio < 1, the penalty is a combination of L1 and L2.

XGBoost[9,10]

XGBoost (eXtreme Gradient Boosting) is a gradient boosting decision tree algorithm that can include higher-order interactions and account for complex nonlinear relationships of variables. Boosting is an ensemble technique where new models are added to correct the errors made by existing models. Models are added sequentially until no further improvements can be made. Gradient boosting is an approach where new models are created that predict the residuals or errors of prior models and then added together to make the final prediction, using a gradient descent algorithm to minimize the loss when adding new models. This approach supports both regression and classification predictive modeling problems, including hazard risk prediction. XGBoost handles sparse data and enables quicker model exploration, and often achieves higher accuracy than a single decision tree.

In our study, XGBoost was applied in Cox proportional hazards model, using the Python package of *xgboost*.[11] The key parameters include:

- objective: Learning objective.
    - survival:cox: Cox regression for right censored survival time data
- eval_metric: Evaluation metrics for validation data
    - cox-nloglik: negative partial log-likelihood for Cox proportional hazards regression
- n_estimators (range: (0,∞], default: 100): The number of trees (or rounds)
- learning_rate (range: [0,1], default: 0.3): Step size shrinkage used in update to prevents overfitting.
- max_depth (range: [0,∞], default: 6): Maximum depth of a tree. Increasing this value will make the model more complex and more likely to overfit. 0 indicates no limit on depth.
- subsample (range: (0,1], default: 1): Subsample ratio of the training instances. Setting it to 0.5 means that XGBoost would randomly sample half of the training data prior to growing trees. and this will prevent overfitting. Subsampling will occur once in every boosting iteration.
- colsample_bytree (range: (0,1], default: 1): Subsample ratio of columns when constructing each tree. Subsampling occurs once for every tree constructed.
- min_child_weight (range: [0,∞], default: 1): Minimum sum of instance weight (hessian) needed in a child. If the tree partition step results in a leaf node with the sum of instance weight less than min_child_weight, then the building process will give up further partitioning. The larger min_child_weight is, the more conservative the algorithm will be.
- reg_lambda (default: 1): L2 regularization term on weights. Increasing this value will make model more conservative.
- reg_alpha (default: 0): L1 regularization term on weights. Increasing this value will make model more conservative.

BorutaSHAP[12]

SHAP (SHapley Additive exPlanations) is a unified approach to explain how much each factor in a model has contributed to the prediction, in other words, it measures the impact in model predictions with and without a particular feature. BorutaSHAP is a wrapper feature selection method, which combines both the Boruta feature selection algorithm with shapley values. This combination has proven to outperform the original Permutation Importance method in both speed, and the quality of the feature subset produced. Not only does this algorithm provide a better subset of features, but it can also simultaneously provide the most accurate and consistent global feature rankings, which can be used for model inference too. BorutaSHAP allows the user to choose any Tree Based learner as the base model in the feature selection process.

In our study, BorutaSHAP was applied in XGBoost survival model, using the Python package of *BorutaShap*,[13] The key parameters include:

- importance_measure ("shap", "gain"or "permutation", default: "shap"): BorutaShap object
- n_trials (range: (0,∞], default: 100): Number of iterations for Boruta algorithm

Assessment of prediction performance

Discrimination: The ability of a model to separate cases from controls

Harrell's C-index: Goodness of fit measure to evaluate risk models in survival analysis. It measures the probability that a randomly selected subject with shorter time-to-event will have a higher predicted probability of event than a randomly selected subject with longer time-to-event.

Reclassification: The ability of a new model to improve on an old model

Integrated discrimination improvement (IDI): It summarises the extent a new model increases risk in events and decreases risk in non-event compared with the old model. ($\overline{\hat{P}}$ represents the average predicted probability for that group)

- IDI = ($\overline{\hat{P}}_{\text{new,events}}$ - $\overline{\hat{P}}_{\text{old,events}}$) – ($\overline{\hat{P}}_{\text{new,non-events}}$ - $\overline{\hat{P}}_{\text{old,non-events}}$)

Net reclassification improvement (NRI): It quantifies the appropriateness of the change in predicted probabilities or categorised risk group when changing from old to new model ($\hat{P}$ represents the proportion and D the occurrence of death)

- Continuous NRI = Continuous $\text{NRI}_{\text{event}}$ + Continuous $\text{NRI}_{\text{non-event}}$
  Continuous $\text{NRI}_{\text{event}}$ = $\hat{P}_{\text{higher predicted prob, D=1}}$ - $\hat{P}_{\text{lower predicted prob, D=1}}$
  Continuous $\text{NRI}_{\text{non-event}}$ = $\hat{P}_{\text{lower predicted prob, D=0}}$ - $\hat{P}_{\text{higher predicted prob, D=0}}$
- Categorical NRI = Categorical $\text{NRI}_{\text{event}}$ + Categorical $\text{NRI}_{\text{non-event}}$
  Categorical $\text{NRI}_{\text{event}}$ = $\hat{P}_{\text{higher risk group, D=1}}$ - $\hat{P}_{\text{lower risk group, D=1}}$
  Categorical $\text{NRI}_{\text{non-event}}$ = $\hat{P}_{\text{lower risk group, D=0}}$ - $\hat{P}_{\text{higher risk group, D=0}}$

Calibration: How close the predicted probability is to the actual (observed) risk

Calibration plot: It reflects how close the predicted probability is to the actual risk in each decile group of predicted probability.

Reference

1. Hippisley-Cox J, Coupland C, Brindle P. Development and validation of QRISK3 risk prediction algorithms to estimate future risk of cardiovascular disease: prospective cohort study. *BMJ (Clinical research ed).* 2017;357:j2099.
2. Parsons RE, Colopy GW, Clifton DA, Clifton L. Clinical Prediction Models in Epidemiological Studies: Lessons from the Application of QRISK3 to UK Biobank Data. *Journal of Data Science.* 2022;20(1):1-13.
3. SCORE2 risk prediction algorithms: new models to estimate 10-year risk of cardiovascular disease in Europe. *European heart journal.* 2021;42(25):2439-2454.
4. Moons KG, Altman DG, Reitsma JB, et al. Transparent Reporting of a multivariable prediction model for Individual Prognosis or Diagnosis (TRIPOD): explanation and elaboration. *Annals of internal medicine.* 2015;162(1):W1-73.
5. Julkunen H, Cichońska A, Tiainen M, et al. Atlas of plasma NMR biomarkers for health and disease in 118,461 individuals from the UK Biobank. *Nature communications.* 2023;14(1):604.
6. Simon N, Friedman J, Hastie T, Tibshirani R. Regularization Paths for Cox's Proportional Hazards Model via Coordinate Descent. *Journal of statistical software.* 2011;39(5):1-13.
7. Zou H, Hastie T. Regularization and variable selection via the elastic net. *Journal of the Royal Statistical Society: Series B (Statistical Methodology).* 2005;67(2):301-320.
8. Clarke R, Emberson JR, Breeze E, et al. Biomarkers of inflammation predict both vascular and non-vascular mortality in older men. *European heart journal.* 2008;29(6):800-809.
9. Chen T, Guestrin C. XGBoost: A Scalable Tree Boosting System. KDD '16: Proceedings of the 22nd ACM SIGKDD International Conference on Knowledge Discovery and Data Mining; 2016; San Francisco, California, USA.
10. Brownlee J. A Gentle Introduction to XGBoost for Applied Machine Learning. 2016; https://machinelearningmastery.com/gentle-introduction-xgboost-applied-machine-learning/.
11. Plummer M. Improved estimates of floating absolute risk. *Statistics in medicine.* 2004;23(1):93-104.
12. Keany E. BorutaShap : A wrapper feature selection method which combines the Boruta feature selection algorithm with Shapley values. *Zenodo.* 2020.
13. Lewington S, Clarke R, Qizilbash N, Peto R, Collins R. Age-specific relevance of usual blood pressure to vascular mortality: a meta-analysis of individual data for one million adults in 61 prospective studies. *Lancet (London, England).* 2002;360(9349):1903-1913.

Table S1: List of clinically validated metabolites for main analyses

	Clinically validated metabolites*	Abbreviation
	**Cholesterols**, mmol/L	
1	Total cholesterol	Total_C
2	VLDL cholesterol	VLDL_C
3	LDL cholesterol	LDL_C
4	HDL cholesterol	HDL_C
	**Triglycerides**, mmol/L	
5	Total triglycerides	Total_TG
	**Fatty acids**, mmol/L	
6	Total fatty acids	TotFA
7	Omega-3 fatty acids	FAw3
8	Omega-6 fatty acids	FAw6
9	Polyunsaturated fatty acids	PUFA
10	Monounsaturated fatty acids	MUFA
11	Saturated fatty acids	SFA
12	Docosahexenoic acid	DHA
13	Linoleic acid	LA
	**Fatty acids ratios**	
14	Omega-3 fatty acids to total fatty acids	FAw3_FA
15	Omega-6 fatty acids to total fatty acids	FAw6_FA
16	Polyunsaturated fatty acids to total fatty acids	PUFA_FA
17	Monounsaturated fatty acids to total fatty acids	MUFA_FA
18	Saturated fatty acids to total fatty acids	SFA_FA
19	Docosahexaenoic acid to total fatty acids	DHA_FA
20	Linoleic acid to total fatty acids	LA_FA
21	Polyunsaturated to monounsaturated fatty acids	PUFA_MUFA
22	Omega-6 fatty acids to omega-3 fatty acids	FAw6_FAw3
	**Apolipoproteins**	
23	Apolipoprotein B, g/l	ApoB
24	Apolipoprotein A1, g/l	ApoA1
25	Apolipoprotein B ratio to apolipoprotein A1	ApoB_ApoA1
	**Amino acids**, mmol/L	
26	Alanine	Ala
27	Glycine	Gly
28	Histidine	His
	**Branched-chain amino acids**, mmol/L	
29	Isoleucine	Ile
30	Leucine	Leu
31	Valine	Val
32	Total concentration of branched-chain amino acids	BCAA
	**Aromatic amino acids**, mmol/L	
33	Phenylalanine	Phe
34	Tyrosine	Tyr
	**Glycolysis related metabolites**, mmol/L	
35	Glucose	Glc
36	Lactate	Lac
	**Fluid balance**	
37	Creatinine, mmol/L	Crea
38	Albumin, g/L	Alb
	**Inflammation**, mmol/L	
39	Glycoprotein acetyls	GlycA

*Clinically and analytically validated biomarkers, which are comparable with other clinically and analytically validated laboratory method, such as photometric or enzymatic methods.

Table S2: List of metabolites for sensitivity analyses

Biomarker name	Abbreviation	Biomarker name	Abbreviation
**Total lipids**, mmol/L		**Fatty acids (concentration), mmol/L**	
Total cholesterol*	Total_C	Polyunsaturated fatty acids*	PUFA
VLDL cholesterol*	VLDL_C	Monounsaturated fatty acids*	MUFA
IDL cholesterol	IDL_C	Saturated fatty acids*	SFA
LDL cholesterol*	LDL_C	Docosahexaenoic acid*	DHA
HDL cholesterol*	HDL_C	Linoleic acid*	LA
Total cholesterol minus HDL-C	non_HDL_C	Omega-3 fatty acids*	FAw3
Remnant cholesterol	Remnant_C	Omega-6 fatty acids*	FAw6
Total esterified cholesterol	Total_CE	Total fatty acids*	TotFA
Total free cholesterol	Total_FC	**Fatty acids ratio, %**	
Total phospholipids	Total_PL	Polyunsaturated fatty acids to total*	PUFA_FA
Total triglycerides*	Total_TG	Monounsaturated fatty acids to total*	MUFA_FA
**Lipoprotein particle concentration, mmol/L**		Saturated fatty acids to total*	SFA_FA
Chylomicrons&extremely large VLDL	XXL_VLDL_P	Docosahexaenoic acid to total*	DHA_FA
Very large VLDL	XL_VLDL_P	Linoleic acid to total*	LA_FA
Large VLDL	L_VLDL_P	Omega-3 fatty acids to total*	FAw3_FA
Medium VLDL	M_VLDL_P	Omega-6 fatty acids to total*	FAw6_FA
Small VLDL	S_VLDL_P	Polyunsaturated to monounsaturated fatty acids*	PUFA_MUFA
Very small VLDL	XS_VLDL_P	Omega-6 to omega-3 fatty acids*	FAw6_FAw3
Total VLDL	VLDL_P	**Cholines, mmol/L**	
IDL	IDL_P	Total cholines	TotCho
Large LDL	L_LDL_P	Phosphatidylcholine	PC
Medium LDL	M_LDL_P	Sphingomyelins	SM
Small LDL	S_LDL_P	Phosphoglycerides	Phosphoglyc
Total LDL	LDL_P	**Amino acids, mmol/L**	
Very large HDL	XL_HDL_P	Alanine*	Ala
Large HDL	L_HDL_P	Glutamine	Gln
Medium HDL	M_HDL_P	Glycine*	Gly
Small HDL	S_HDL_P	Histidine*	His
Total HDL	HDL_P	Isoleucine*	Ile
**Mean lipoportein particle size, nm**		Leucine*	Leu
VLDL	VLDL_D	Valine*	Val
LDL	LDL_D	Branched-chain amino acids*	BCAA
HDL	HDL_D	Phenylalanine*	Phe
**Lipoprotein particle composition**		Tyrosine*	Tyr
Esterified cholesterol in VLDL	VLDL_CE	**Glycolysis related metabolites, mmol/L**	
Free cholesterol in VLDL	VLDL_FC	Lactate*	Lac
Phospholipids in VLDL	VLDL_PL	Citrate	Cit
Triglycerides in VLDL	VLDL_TG	Glucose*	Glc
Esterified cholesterol in IDL	IDL_CE	Pyruvate	Pyruvate
Free cholesterol in IDL	IDL_FC	**Ketone bodies, mmol/L**	
Phospholipids in IDL	IDL_PL	Acetate	Ace
Triglycerides in IDL	IDL_TG	Aceto acetate	AcAce
Esterified cholesterol in LDL	LDL_CE	Acetone	Acetone
Free cholesterol in LDL	LDL_FC	Beta-hydroxybutyrate	bOHBut
Phospholipids in LDL	LDL_PL	**Fluid balance**	
Triglycerides in LDL	LDL_TG	Albumin*, g/L	Alb
Esterified cholesterol in HDL	HDL_CE	Creatinine*, mmol/L	Crea_nmr
Free cholesterol in HDL	HDL_FC	**Inflammation, mmol/L**	
Triglycerides in HDL	HDL_TG	Glycoprotein acetyls*	Gp
Phospholipids in HDL	HDL_PL		
**Apolipoproteins, g/L**			
Apolipoprotein A-I*	ApoA-1		
Apolipoprotein B*	ApoB		
Apolipoprotein B to A-1 ratio*	ApoB_ApoA-1		

*The clinical-validated metabolites used in the main analyses

Table S3: Disease and medication codes of QRISK3 variables in UK biobank

	ICD-10 code	Verbal interview or questionnaire code	Medication code or other measurement
Diabetes			
Type 1	E10	1222; Variable ID 2443=1 & age≤20	If recode both type1 & type2, then categorize as type1
Type 2	E11;E13;E14	1220;1223; Variable ID 2443=1 & age>20	HbA1c≥48 & ≤184 mmol/mol
Chronic kidney disease (stage 3, 4, 5)	N183; N184; N185; N180	1193	eGFR <60 ml/min
Atrial fibrillation	I48	1471;1483	
Hypertension treatment		Variable ID 6177, 6153 =2	1140860192, 1140860292, 1140860696, 1140860728, 1140860750, 1140860806, 1140860882, 1140860904, 1140861088, 1140861190, 1140861276, 1140866072, 1140866078, 1140866090, 1140866102, 1140866108, 1140866122, 1140866138, 1140866156, 1140866162, 1140866724, 1140866738, 1140868618, 1140872568, 1140874706, 1140874744, 1140875808, 1140879758, 1140879760, 1140879762, 1140879802, 1140879806, 1140879810, 1140879818, 1140879822, 1140879826, 1140879830, 1140879834, 1140879842, 1140879866, 1140884298, 1140888552, 1140888556, 1140888560, 1140888646, 1140909706, 1140910442, 1140910614, 1140916356, 1140923272, 1140923336, 1140923404, 1140923712, 1140926778, 1140928226, 1141145660, 1141146126, 1141152998, 1141153026, 1141164276, 1141165470, 1141166006, 1141169516, 1141171336, 1141180592, 1141180772, 1141180778, 1141184722, 1141193282, 1141194794, 1141194810
Migraines	G43	1265	
Rheumatoid arthritis	M05; M06	1464	
Systemic lupus erythematosus	M32	1381	
Severe mental illness*	F20; F31; F331; F332; F333	1289;1291; Variable ID 20126=1,2,3,4	
Atypical antipsychotic medication			1140867420, 1140867444, 1140927956, 1140928916, 1141152848, 1141153490, 1141169714, 1141195974
Regular steroid tablets			1140874790, 1140874816, 1140874896, 1140874930, 1140874976, 1141145782, 1141173346
Erectile disfunction	N484	1518	1141168936, 1141168948, 1141168944, 1141168946, 1140869100, 1140883010

* Includes schizophrenia, bipolar disorder and moderate/severe depression.

Table S4: ICD-10 and operation code of cardiovascular disease

ICD/OPCS category	Disease category	Code definition
I20	Angina pectoris	I20.0 Unstable angina<br>I20.1 Angina pectoris with documented spasm<br>I20.8 Other forms of angina pectoris<br>I20.9 Angina pectoris, unspecified angina
I21	Acute myocardial infarction	I21.0 Acute transmural myocardial infarction of anterior wall<br>I21.1 Acute transmural myocardial infarction of inferior wall<br>I21.2 Acute transmural myocardial infarction of other sites<br>I21.3 Acute transmural myocardial infarction of unspecified site<br>I21.4 Acute subendocardial myocardial infarction<br>I21.9 Acute myocardial infarction, unspecified
I22	Subsequent myocardial infarction	I22.0 Subsequent myocardial infarction of anterior wall<br>I22.1 Subsequent myocardial infarction of inferior wall<br>I22.8 Subsequent myocardial infarction of other sites<br>I22.9 Subsequent myocardial infarction of unspecified site
I23	Certain current complications following acute myocardial infarction	I23.0 Haemopericardium as current complication following acute myocardial infarction;<br>I23.1 Atrial septal defect as current complication following acute myocardial infarction;<br>I23.2 Ventricular septal defect as current complication following acute myocardial infarction;<br>I23.3 Rupture of cardiac wall without haemopericardium as current complication following acute myocardial infarction;<br>I23.4 Rupture of chordae tendineae as current complication following acute myocardial infarction<br>I23.5 Rupture of papillary muscle as current complication following acute myocardial infarction<br>I23.6 Thrombosis of atrium, auricular appendage, and ventricle as current complications following acute myocardial infarction;<br>I23.8 Other current complications following acute myocardial infarction
I24	Other acute ischaemic heart diseases	I24.0 Coronary thrombosis not resulting in myocardial infarction<br>I24.1 Dressler’s syndrome<br>I24.8 Other forms of acute ischaemic heart disease<br>I24.9 Acute ischaemic heart disease, unspecified (excl. ischaemic heart disease (chronic) NOS)
I25	Chronic ischaemic heart disease	I25.0 Atherosclerotic cardiovascular disease, so described<br>I25.1 Atherosclerotic heart disease<br>I25.2 Old myocardial infarction<br>I25.3 Aneurysm of heart<br>I25.4 Coronary artery aneurysm<br>I25.5 Ischaemic cardiomyopathy<br>I25.6 Silent myocardial ischaemia<br>I25.8 Other forms of chronic ischaemic heart disease - Any condition in I21-I22 and I24.- specified as chronic<br>I25.9 Chronic ischaemic heart disease, unspecified - Ischaemic heart disease (chronic) NOS
I63	Cerebral infarction	I63.0 Cerebral infarction due to thrombosis of precerebral arteries<br>I63.1 Cerebral infarction due to embolism of precerebral arteries<br>I63.2 Cerebral infarction due to unspecified occlusion or stenosis of precerebral arteries<br>I63.3 Cerebral infarction due to thrombosis of cerebral arteries<br>I63.4 Cerebral infarction due to embolism of cerebral arteries<br>I63.5 Cerebral infarction due to unspecified occlusion or stenosis of cerebral arteries

		I63.6 Cerebral infarction due to cerebral venous thrombosis, nonpyogenic<br>I63.8 Other cerebral infarction<br>I63.9 Cerebral infarction, unspecified
I64	Stroke	Stroke, not specified as haemorrhage or infaction
G45	Transient cerebral ischaemic attacks	G45.0 Vertebro-basilar artery syndrome<br>G45.1 Carotid artery syndrome (hemispheric)<br>G45.2 Multiple and bilateral precerebral artery syndromes<br>G45.3 Amaurosis fugax<br>G45.4 Transient global amnesia<br>G45.8 Other transient cerebral ischaemic attacks and related syndromes<br>G45.9 Transient cerebral ischaemic attack, unspecified
K40		Saphenous vein graft replacement of coronary artery
K41		Other autograft replacement of coronary artery
K42		Allograft replacement of coronary artery
K43		Prosthetic replacement of coronary artery
K44		Other replacement of coronary artery
K45		Connection of thoracic artery to coronary artery
K46		Other bypass of coronary artery
K47		Repair of coronary artery
K49		Transluminal balloon angioplasty of coronary artery
K50		Other therapeutic transluminal operations on coronary artery
K75		Percutaneous transluminal balloon angioplasty and insertion of stent into coronary artery

Figure S1: Flowchart of exclusion criteria for study population in UK Biobank

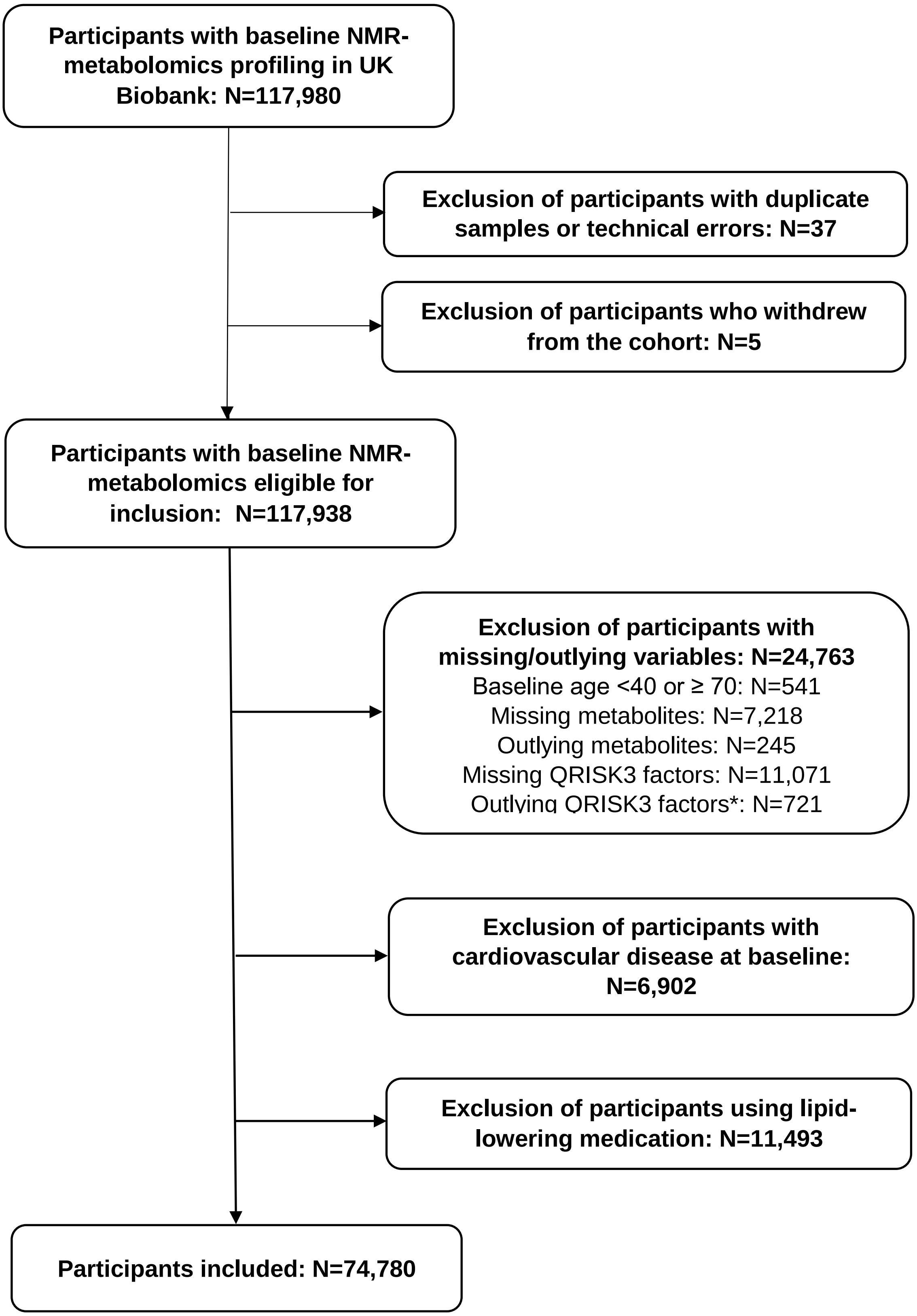

*****Exclude outlying of baseline standard deviation of systolic blood pressure>20, most missing in QRISK3 variables come from clinical chemistry measurement of total cholesterol to HDL cholesterol ratio (n=10,794)

Table S5: Fine-tuning of hyper-parameters (QRISK3)

Model	Package (Python)	Hyperparameter*	Tuning Range	Tuning Step†	Selected Value	
					Women	Men
Model 1	CoxPHSurvivalAnalysis	-	-	-		
Model 2	CoxnetSurvivalAnalysis (Penalized Cox Model)	L1_ratio	(0.7, 1.0)	0.05	0.9	0.9
		alphas	alpha_min_ratio=0.01, max_iter=1000 to search for 1000 α values up to 1% of estimated maximum.		0.00041	0.0012
Model 3	XGBoost	objective	-	-	survival:cox	
		eval_metric	-	-	cox-nloglik	
		learning_rate	[0.01, 0.02, 0.05, 0.1]		0.01	0.01
		max_depth	(2,5)	1	4	4
		n_estimators	(50,2000)	50	550	450
		subsample	(0.5, 1.0)	0.1	0.8	0.6
		colsample_bytree	(0.5, 1.0)	0.1	0.5	0.9
		min_child_weight	(6,30)	1	21	18
		reg_lambda	Start from [1e-6, 1e-4, 0.01, 0.1, 1, 10, 100]		2.3	1.6
		reg_alpha	Start from [0, 1e-6, 1e-4, 0.01, 0.1, 1, 10, 100]		0.18	0.0036
	BorutaShap	importance_measure	-	-	SHAP	
		n_trials	-	-	100	
	CoxPHSurvivalAnalysis	-	-	-		

*The meaning of each hyperparameter is explained in eMethods; † Tuning the hyperparameter from the lowest value to the highest value in the tuning range, with increase of the tuning step each time, and selected the hyperparmeter with the best performance.

Table S6: Baseline characteristics in UK Biobank versus in study population

	UK Biobank population		Study Population	
	**Women**	**Men**	**Women**	**Men**
**No. of participants**	232,744	169,405	42,427	32,353
**Age and socioeconomic factors**				
Baseline Age, years	55.5 (8.0)	55.3 (8.2)	55.4 (7.9)	55.1 (8.2)
White, %	94.4	94.1	95.0	94.8
Townsend deprivation index*	-1.4 (3.0)	-1.3 (3.1)	-1.5 (3.0)	-1.4 (3.0)
**Anthropometry, blood pressure, and lipids by clinical chemistry**				
Body Mass Index, kg/m^2	26.7 (5.0)	27.4 (4.0)	26.7 (4.9)	27.4 (4.0)
Systolic blood pressure, mmHg	134.4 (19.2)	140.3 (17.3)	134.0 (18.9)	140.2 (17.2)
Standard deviation between two readings†, mmHg	5.4 (4.5)	5.2 (4.3)	5.2 (4.1)	5.0 (3.9)
Total cholesterol to HDL-C ratio	3.9 (1.0)	4.6 (1,1)	3.9 (1.0)	4.6 (1.1)
**Smoking intensity**, %				
Ex-smoker	30.7	34.9	30.9	35.0
Light smoker (< 10 per day)	3.9	6.1	3.9	5.9
Moderate smoker (10-19 per day)	3.0	3.1	3.0	3.0
Heavy smoker (≥20 per day)	1.9	3.4	1.8	3.3
**Family history of heart disease**‡, %	42.8	36.1	43.2	36.3
Parents	40.1	33.7	40.5	33.9
Siblings	7.2	5.4	7.2	5.3
**Disease and medication history**, %				
Type 1 diabetes	0.5	0.2	0.5	0.2
Type 2 diabetes	1.3	2.4	1.3	2.3
Chronic kidney disease	1.7	1.1	1.8	1.2
Atrial fibrillation	0.6	1.4	0.6	1.3
Migraines	6.0	2.2	6.3	2.3
Rheumatoid arthritis	1.5	0.8	1.5	0.8
Systemic lupus erythematosus	0.2	<0.1	0.2	<0.1
Severe mental illness§	0.5	0.5	5.9	3.8
Erectile dysfunction	-	0.5	-	0.6
Hypertension treatment	12.5	12.6	12.2	12.5
Atypical antipsychotic medication	0.2	0.3	0.2	0.3
Regular steroid tablets	0.8	0.7	0.8	0.8

Characteristics of QRISK factors at baseline by sex. Continuous variables are presented as mean (standard deviation) and categorical variables are presented as column percentages. *Higher values indicate higher levels of material deprivation; †QRISK asks for standard deviation of systolic blood pressure values recorded in the five years before study entry, but UK biobank only provided two automated or manual readings at study entry; ‡QRISK asks for the family history in first degree relatives aged less than 60 years, but UK biobank only identified family history in first degree relatives in all ages; §Includes schizophrenia, bipolar disorder and moderate/severe depression. HDL-C=high-density lipoproteins cholesterol.

Table S7: List of selected novel metabolites by different methods (QRISK3)

Clinically validated metabolites	Women				Men			
	Significant associated*	Independent associated†	Elestic -net‡	Boruta SHAP¶	Significant associated	Independent associated	Elestic -net	Boruta SHAP
**Cholesterols&Triglycerides**								
Total_C					✓			
VLDL_C	✓				✓			✓
LDL_C	✓			✓	✓			
HDL_C	✓	✓			✓		✓	
Total_TG	✓		✓	✓	✓		✓	
**Fatty acids**								
Total FA	✓				✓			
Omega-3 FA			✓			✓	✓	
Omega-6 FA					✓			
PUFA					✓			
MUFA	✓				✓			
SFA	✓				✓			
DHA								
LA					✓			
Omega-3 FA to total FA					✓	✓	✓	
Omega-6 FA to total FA	✓	✓		✓				
PUFA to total FA	✓	✓			✓			
MUFA to total FA	✓	✓		✓	✓		✓	
SFA to total FA				✓				✓
DHA to total FA	✓	✓			✓			
LA to total FA	✓	✓	✓					
PUFA to MUFA	✓	✓	✓		✓			
Omega-6 to omega-3 FA			✓					✓
**Apolipoproteins**								
ApoB	✓		✓		✓			
ApoA-1	✓	✓			✓			
ApoB to ApoA-1	✓	✓		✓	✓	✓	✓	✓
**Amino acids**								
Alanine							✓	
Glycine	✓		✓		✓		✓	✓
Histidine	✓	✓	✓					
Isoleucine								
Leucine							✓	✓
Valine			✓				✓	
BACC								
Phenylalanine	✓		✓		✓		✓	
Tyrosine				✓				✓
**Glycolysis related**								
Glucose				✓				✓
Lactate							✓	
**Fluid balance**								
Creatinine					✓			
Albumin	✓	✓	✓	✓	✓	✓	✓	✓
**Inflammation**								
Glycoprotein acetyls	✓	✓	✓	✓	✓	✓	✓	✓

*Association was calculated using Cox proportional-hazards regression with adjustment of established risk factors, including age, education, region, townsend deprivation index, smoking, alcohol intake, body mass index, systolic blood pressure, and baseline diabetes; Significant association was defined as p-value<0.01 after correction of false discovery rate using Benjamini-Hochberg method; †Association was calculated using Cox proportional-hazards regression with adjustment of QRISK3 score; ‡Novel metabolites selected by elastic-net based on Cox proportional-hazards regression, when adding all metabolites into the model; ¶ Novel metabolites selected by BorutaSHAP from XGBoost survival model, when adding all metabolites into the model

Table S8. Associations of clinical metabolites independent from SCORE2

	Hazard ratio (95% CI)	
	**Women**	**Men**
**Recalibrated SCORE2**	**1.12 (1.10, 1.13)**	**1.07 (1.06, 1.07)**
**Cholesterols & Triglycerides**		
Total cholesterol	0.96 (0.92, 1.01)	0.98 (0.95, 1.02)
VLDL cholesterol	1.06 (1.01, 1.11)	1.01 (0.97, 1.05)
LDL cholesterol	0.99 (0.95, 1.04)	0.99 (0.96, 1.03)
HDL cholesterol	**0.87 (0.83, 0.92)***	0.95 (0.91, 0.99)
Total triglycerides	1.05 (1.01, 1.10)	0.99 (0.95, 1.03)
**Fatty acids**		
Total fatty acids	1.03 (0.99, 1.08)	0.98 (0.95, 1.02)
Omega-3 fatty acids	0.96 (0.92, 1.01)	**0.93 (0.90, 0.96)***
Omega-6 fatty acids	0.98 (0.94, 1.03)	0.99 (0.95, 1.02)
Polyunsaturated fatty acids	0.97 (0.93, 1.02)	0.97 (0.93, 1.00)
Monounsaturated fatty acids	**1.08 (1.03, 1.12)***	1.00 (0.96, 1.04)
Saturated fatty acids	1.04 (0.99, 1.08)	0.98 (0.95, 1.02)
Docosahexenoic acid	0.94 (0.90, 0.98)	**0.93 (0.90, 0.96)***
Linoleic acid	0.97 (0.93, 1.02)	0.99 (0.95, 1.02)
Omega-3 to total fatty acids	0.94 (0.90, 0.98)	**0.92 (0.89, 0.96)***
Omega-6 to total fatty acids	**0.91 (0.87, 0.95)***	1.01 (0.98, 1.05)
Polyunsaturated to total fatty acids	**0.89 (0.85, 0.93)***	0.98 (0.95, 1.02)
Monounsaturated to total fatty acids	**1.16 (1.11, 1.22)***	1.03 (0.99, 1.07)
Saturated to total fatty acids	1.03 (0.99, 1.08)	1.00 (0.96, 1.04)
Docosahexaenoic acid to total fatty acids	**0.92 (0.88, 0.96)***	**0.93 (0.90, 0.97)***
Linoleic acid to total fatty acids	**0.90 (0.86, 0.94)***	1.00 (0.97, 1.04)
Polyunsaturated to monounsaturated fatty acids	**0.85 (0.81, 0.89)***	0.97 (0.94, 1.01)
Omega-6 to omega-3 fatty acids	1.03 (0.99, 1.08)	**1.06 (1.02, 1.09)***
**Apolipoproteins**		
Apolipoprotein B	1.03 (0.98, 1.08)	1.01 (0.98, 1.05)
Apolipoprotein A-1	**0.90 (0.86, 0.94)***	**0.94 (0.90, 0.97)***
Apolipoprotein B to apolipoproteinA-1	**1.09 (1.04, 1.14)***	**1.07 (1.03, 1.11)***
**Amino acids**		
Alanine	1.03 (0.99, 1.08)	0.98 (0.95, 1.02)
Glycine	0.94 (0.89, 0.98)	0.95 (0.92, 0.99)
Histidine	**0.91 (0.87, 0.95)***	0.97 (0.93, 1.00)
Isoleucine	1.04 (0.99, 1.08)	1.03 (0.99, 1.06)
Leucine	1.02 (0.98, 1.06)	1.01 (0.98, 1.05)
Valine	1.01 (0.97, 1.05)	1.00 (0.96, 1.03)
Total branched-chain amino acids	1.02 (0.98, 1.06)	1.01 (0.97, 1.04)
Phenylalanine	1.06 (1.02, 1.11)	**1.06 (1.03, 1.10)***
Tyrosine	1.02 (0.97, 1.06)	1.02 (0.99, 1.06)
**Glycolysis related metabolites**		
Glucose	1.03 (0.99, 1.07)	1.03 (0.99, 1.06)
Lactate	1.03 (0.99, 1.08)	0.99 (0.95, 1.02)
**Fluid balance**		
Creatinine	1.05 (1.01, 1.09)	1.03 (1.00, 1.06)
Albumin	**0.86 (0.82, 0.90)***	**0.89 (0.86, 0.93)***
**Inflammation**		
Glycoprotein acetyls	**1.18 (1.13, 1.23)***	**1.08 (1.04, 1.12)***

Hazard ratios (HR) per one score higher of concentration. HR of each metabolite was calculated by Cox proportional-hazards regression with adjustment of SCORE2. *Associations remained significant (p-value<0.01) by correction of false discovery rate using Benjamini-Hochberg method.

Table S9: List of selected metabolites using different methods (SCORE2)

Clinically validated metabolites	Women				Men			
	Significant associated*	Independent associated†	Elestic -net‡	Boruta SHAP¶	Significant associated	Independent associated	Elestic -net	Boruta SHAP
**Cholesterols&Triglycerides**								
Total_C					✓			
VLDL_C	✓				✓			✓
LDL_C	✓			✓	✓			
HDL_C	✓	✓			✓			
Total_TG	✓		✓	✓	✓		✓	
**Fatty acids**								
Total FA	✓				✓			
Omega-3 FA			✓			✓	✓	
Omega-6 FA					✓			
PUFA					✓			
MUFA	✓	✓			✓			
SFA	✓				✓			
DHA						✓		
LA					✓			
Omega-3 FA to total FA					✓	✓		
Omega-6 FA to total FA	✓	✓		✓				
PUFA to total FA	✓	✓			✓			
MUFA to total FA	✓	✓		✓	✓		✓	
SFA to total FA				✓				✓
DHA to total FA	✓	✓			✓	✓		
LA to total FA	✓	✓	✓					
PUFA to MUFA	✓	✓	✓		✓			
Omega-6 to omega-3 FA			✓			✓		✓
**Apolipoproteins**								
ApoB	✓		✓		✓			
ApoA-1	✓	✓			✓	✓		
ApoB to ApoA-1	✓	✓	✓	✓	✓	✓	✓	✓
**Amino acids**								
Alanine							✓	
Glycine	✓		✓		✓		✓	✓
Histidine	✓	✓	✓					
Isoleucine								
Leucine								✓
Valine			✓				✓	
BACC								
Phenylalanine	✓		✓		✓	✓	✓	
Tyrosine			✓	✓			✓	✓
**Glycolysis related**								
Glucose			✓	✓				✓
Lactate			✓				✓	
**Fluid balance**								
Creatinine			✓		✓		✓	
Albumin	✓	✓	✓	✓	✓	✓	✓	✓
**Inflammation**								
Glycoprotein acetyls	✓	✓	✓	✓	✓	✓	✓	✓

*Association was calculated using Cox proportional-hazards regression with adjustment of established risk factors, including age, education, region, townsend deprivation index, smoking, alcohol intake, body mass index, systolic blood pressure, and baseline diabetes; Significant association was defined as p-value<0.01 after correction of false discovery rate using Benjamini-Hochberg method; †Association was calculated using Cox proportional-hazards regression with adjustment of SCORE2; ‡ Novel metabolites selected by elastic-net based on Cox proportional-hazards regression, when adding all metabolites into the model; ¶ Novel metabolites selected by BorutaSHAP from XGBoost survival model, when adding all metabolites into the model.

Table S10: Comparing prediction performance of 10-year CVD risk w/o metabolites (SCORE2)

Prediction Performance	Women (95% CI*)	Men (95% CI)
**Recalibrated SCORE2**		
Harrell's C-index †	0.731 (0.718, 0.744)	0.689 (0.679, 0.699)
**Adding metabolites associated with CVD independently from SCORE2**		
Harrell's C-index	0.745 (0.732, 0.758)	0.695 (0.686, 0.705)
IDI‡ (%)	0.39 (0.24, 0.52)	0.34 (0.20, 0.44)
Continuous NRI§ (%)	21.1 (15.7, 26.3)	15.3 (11.3, 19.3)
events	9.2 (3.9, 14.3)	6.2 (2.3, 10.0)
non-events	12.0 (11.1, 12.9)	9.1 (8.0, 10.2)
Categorical NRI (%)	1.5 (-0.1, 2.8)	0.4 (-1.0, 1.8)
events	2.3 (0.8, 3.7)	-0.2 (-1.5, 1.2)
non-events	-0.9 (-1.0, -0.7)	0.6 (0.3, 0.8)
**Adding metabolites with regularization (using Elastic-net)**		
Harrell's C-index	0.746 (0.734, 0.758)	0.695 (0.685, 0.705)
IDI (%)	0.36 (0.20, 0.49)	0.21 (0.10, 0.30)
Continuous NRI (%)	20.1 (14.5, 25.1)	7.6 (3.3,11.6)
events	7.5 (2.0,12.3)	5.1 (0.6, 9.0)
non-events	12.7 (11.7, 13.6)	2.5 (1.4, 3.6)
Categorical NRI (%)	1.4 (-0.1, 3.0)	0.2 (-1.2, 1.6)
events	2.3 (0.8, 3.8)	-0.4 (-1.7, 1.0)
non-events	-0.9 (-1.0, -0.7)	0.6 (0.3, 0.9)
**Adding metabolites selected by BorutaSHAP from XGBoost**		
Harrell's C-index	0.747 (0.734, 0.758)	0.694 (0.685, 0.704)
IDI (%)	0.36 (0.21, 0.48)	0.27 (0.14, 0.36)
Continuous NRI (%)	21.9 (16.1, 27.3)	13.3 (9.1 17.7)
events	5.2 (-0.4, 10.4)	2.9 (-1.2, 6.8)
non-events	16.7 (15.7, 17.6)	10.4 (9.2, 11.5)
Categorical NRI (%)	1.4 (0, 2.8)	0.5 (-0.8, 1.7)
events	2.2 (0.9, 3.7)	-0.1 (-1.4, 1.0)
non-events	-0.8 (-1.0, -0.7)	0.6 (0.3, 0.9)

Comparing prediction performance of 10-year CVD risk w/o metabolites. In all models, metabolites are added to recalibrated SCORE2 using Cox proportional-hazards regression. Hyper-parameters of each model are in appendix. *Bootstrap percentile confidence interval, bootstrap for 500 times; †Harrell's C-index, measuring the probability that a randomly selected subject with shorter time-to-event will have a higher predicted probability of event than a randomly selected subject with longer time-to-event; ‡Integrated discrimination improvement, summarising the extent a new model increases risk in events and decreases risk in non-event compared with the old model; §Net reclassification improvement, quantifying the appropriateness of the change in predicted probabilities or categorised risk group when changing from old to new model; Categorical NRI is based on a 10% risk threshold.

Figure S2: Calibration of risk prediction models for 10-year CVD risk (SCORE2)

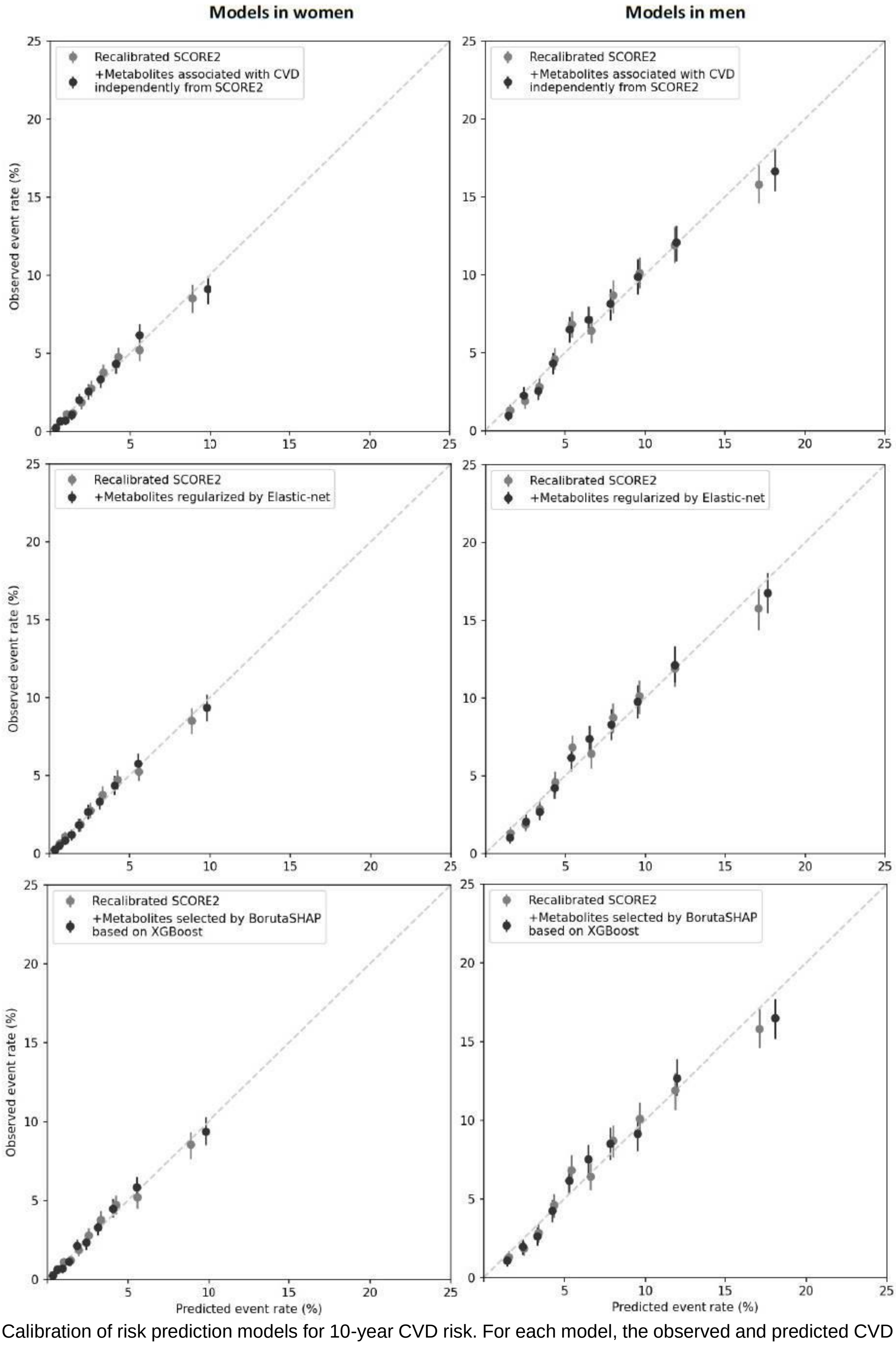

Calibration of risk prediction models for 10-year CVD risk. For each model, the observed and predicted CVD event rates are shown for each of 10 equally sized groups of absolute predicted risk. Vertical lines represent 95% CIs (bootstrap percentile confidence interval, bootstrap for 500 times).

Table S11: Prediction performance of 10-year ASCVD risk w/o metabolites (QRISK3 and wider scope of candidate metabolites)

Prediction Performance	Women (95% CI*)	Men (95% CI)
**Recalibrated QRISK3**		
Harrell's C-index †	0.750 (0.739, 0.763)	0.706 (0.696, 0.716)
**Adding metabolites associated with CVD independently from QRISK3 score**		
Harrell's C-index	0.759 (0.748, 0.770)	0.712 (0.702, 0.722)
IDI‡ (%)	0.49 (0.21, 0.65)	0.31 (0.18, 0.40)
Continuous NRI§ (%)	17.3 (11.6, 22.2)	10.0 (5.5,13.8)
events	7.1 (1.4, 12.2)	1.6 (-2.6, 5.2)
non-events	10.2 (9.3, 11.2)	8.4 (7.3, 9.6)
Categorical NRI (%)	1.5 (-0.2, 3.0)	0.8 (-0.7, 2.2)
events	2.3 (0.6, 3.8)	0.2 (-1.3, 1.6)
non-events	-0.8 (-1.0, -0.7)	0.6 (0.3, 0.9)
**Adding metabolites with regularization (using Elastic-net)**		
Harrell's C-index	0.760 (0.749, 0.772)	0.711 (0.701, 0.720)
IDI (%)	0.24 (0.08, 0.36)	0.12 (0.01, 0.21)
Continuous NRI (%)	6.7 (1.5, 11.9)	2.8 (-1.5, 7.1)
events	6.7 (1.6, 11.8)	7.2 (3.1, 11.4)
non-events	-0.5 (-1.0, 0.9)	-4.4 (-5.4, -3.3)
Categorical NRI (%)	0.8 (-0.9, 2.2)	0.6 (-0.7, 1.8)
events	1.2 (-0.4, 2.7)	0.1 (-1.2, 1.3)
non-events	-0.5 (-0.6, -0.3)	0.5 (0.2, 0.8)
**Adding metabolites selected by BorutaSHAP from XGBoost**		
Harrell's C-index	0.760 (0.748, 0.771)	0.710 (0.700, 0.720)
IDI (%)	0.35 (0.20, 0.47)	0.19 (0.09, 0.27)
Continuous NRI (%)	17.4 (12.0, 23.7)	9.2 (5.0, 13.6)
events	5.6 (0.4, 11.1)	1.4 (-2.6, 5.4)
non-events	11.8 (10.8, 12.7)	7.8 (6.7, 8.9)
Categorical NRI (%)	0.6 (-0.7, 2.0)	1.0 (-0.2, 2.2)
events	1.3 (-0.1, 2.7)	0.6 (-0.7, 1.7)
non-events	-0.7 (-0.8, -0.5)	0.5 (0.2, 0.7)

Comparing prediction performance of 10-year ASCVD risk w/o metabolites. In all models, metabolites are added to recalibrated QRISK3 using Cox proportional-hazards regression. Hyper-parameters of each model are in appendix. *Bootstrap percentile confidence interval, bootstrap for 500 times; †Harrell's C-index, measuring the probability that a randomly selected subject with shorter time-to-event will have a higher predicted probability of event than a randomly selected subject with longer time-to-event; ‡Integrated discrimination improvement, summarising the extent a new model increases risk in events and decreases risk in non-event compared with the old model; §Net reclassification improvement, quantifying the appropriateness of the change in predicted probabilities or categorised risk group when changing from old to new model; Categorical NRI is based on a 10% risk threshold.

Figure S3: Calibration of risk prediction models for 10-year ASCVD risk (QRISK3 and wider scope of candidate metabolites)

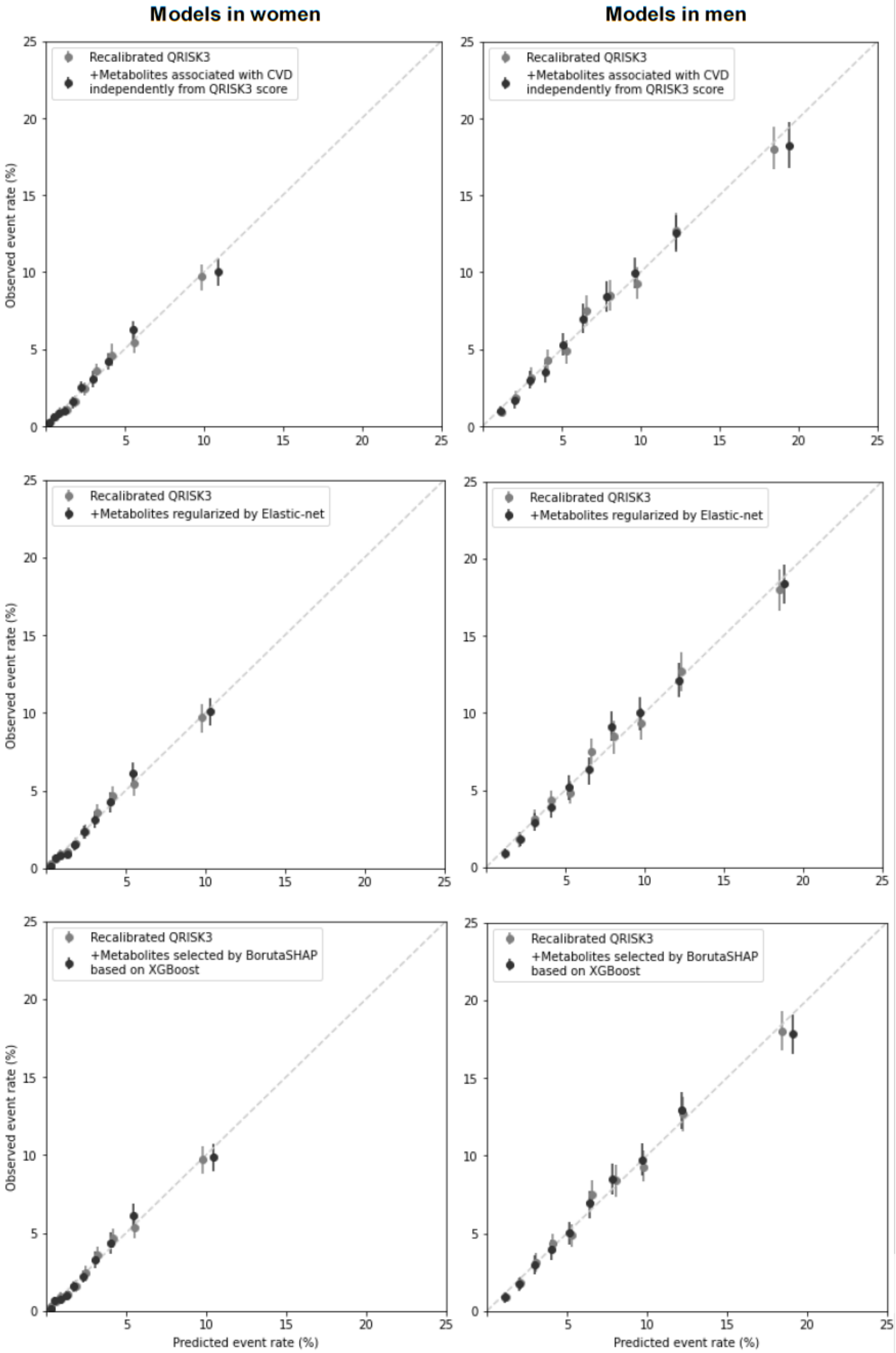

Calibration of risk prediction models for 10-year ASCVD risk. For each model, the observed and predicted CVD event rates are shown for each of 10 equally sized groups of absolute predicted risk. Vertical lines represent 95% CIs (bootstrap percentile confidence interval, bootstrap for 500 times).
